# Predicting Surface Residual Stress for Multi-Axis Milling of Ti-6Al-4V Titanium Alloy in Combined Simulation and Experiments

**DOI:** 10.3390/ma15186471

**Published:** 2022-09-18

**Authors:** Zongyuan Wang, Jinhua Zhou, Junxue Ren, Ailing Shu

**Affiliations:** Laboratory of High Performance Manufacturing for Aero Engine, Ministry of Industry and Information Technology, School of Mechanical Engineering, Northwestern Polytechnical University, Xi’an 710072, China

**Keywords:** surface residual stress, multi-axis milling, RBF neural network, 3D simulation, Ti-6Al-4V titanium alloy

## Abstract

As one essential indicator of surface integrity, residual stress has an important influence on the fatigue performance of aero engines’ thin-walled parts. Larger compressive or smaller tensile residual stress is more prone to causing fatigue cracks. To optimize the state of residual stress, the relationship between the surface residual stress and the machining conditions is studied in this work. A radial basis function (RBF) neural network model based on simulated and experimental data is developed to predict the surface residual stress for multi-axis milling of Ti-6Al-4V titanium alloy. Firstly, a 3D numerical model is established and verified through a cutting experiment. These results are found to be in good agreement with average absolute errors of 11.6% and 15.2% in the σ*_x_* and σ*_y_* directions, respectively. Then, the RBF neural network is introduced to relate the machining parameters with the surface residual stress using simulated and experimental samples. A good correlation is observed between the experimental and the predicted results. The verification shows that the average prediction error rate is 14.4% in the σ*_x_* direction and 17.2% in the σ*_y_* direction. The effects of the inclination angle, cutting speed, and feed rate on the surface residual stress are investigated. The results show that the influence of machining parameters on surface residual stress is nonlinear. The proposed model provides guidance for the control of residual stress in the precision machining of complex thin-walled structures.

## 1. Introduction

Complex curved thin-walled structures using free-form materials have been widely used to improve the performance of final products [1]. Multi-axis machining technology has been used for the finishing of complex curved thin-walled structures due to its excellent accuracy and efficiency [2,3]. Residual stress is inevitably generated during machining, easily causing the deformation of thin-walled parts, and bringing great difficulties to the control of machining quality [4]. Therefore, it is necessary to accurately predict the machining-induced residual stress and determine the optimal residual stress state to improve the fatigue performance of the parts.

In recent decades, many scholars have been exploring methods of predicting machining-induced residual stress. Based on the stress invariance assumption and the stress–strain relaxation method, Li et al. [5] believe that the deformation caused by machining is affected by the magnitude and depth of the initial stress and residual stress. Gao et al. [6] established a semi-analytical model to predict the deformation caused by residual stress in typical machining processes. They found that reducing the residual stress near the blank surface would significantly reduce the machining deformation. Liang et al. [7] proposed a multiphysics prediction model for residual stress considering tool wear. The verification results showed that the surface residual stress changed from compressive stress to tensile stress with the increase in tool wear. Ji et al. [8,9] presented a sensitivity analysis of residual stress based on a verified residual stress prediction model. A parametric study was carried out to investigate the effects of a minimal quantity of lubrication parameters. The results showed that a high maximum compressive residual stress was obtained under a lower flow rate of minimum-quantity lubrication. The advantage of the analytical method is that the residual stress can be quickly calculated, opening up a new and effective approach for the rapid prediction of machining residual stress. However, due to the excessive simplification in the modeling process, there is still a certain distance from engineering applications.

In the past, numerous researchers had worked on numerical modeling of residual stress generation in machining processes. Outeiro et al. [10] numerically analyzed the effects of tool geometry parameters on residual stress during cutting of AISI316L steel. The research showed that increasing the tool edge radius and decreasing the tool rake angle resulted in an increase in residual compressive stress. Arrazola et al. [11] used 3D numerical simulation to predict the residual stress distribution in the cutting of IN718 alloy, and the influence of process parameters on the residual stress distribution was analyzed. They concluded that the surface residual stress and the maximum compressive peak stress both increased with the increase in cutting speed. Xin et al. [12] numerically analyzed the effects of cutting force, cutting temperature, and microstructure on residual stress during disc milling. They discovered that the greater the cutting speed, the smaller the residual compressive stress, and that residual compressive stress changes from compressive stress to tensile stress with the increase in depth. Sahu et al. [13] used 3D numerical simulation to obtain large amounts of simulation data, and the response surface methodology was used to predict the residual stress of turning Ti-6Al-4V. The results showed that the surface residual stress was compressive stress within the threshold of the selected process parameters. Özel and Ulutan [14] presented a 3D numerical model to study the effects of uncoated tools and TiAlN-coated tools on the residual stress of turning Ti-6Al-4V titanium alloy. The research showed that the tool coating had little effect on the circumferential residual stress. However, the coated tools had a greater impact on the peak residual compressive stress compared with the uncoated tools. Mishra et al. [15] simulated the effects of TiAlN-coated tools on cutting temperature and residual stress by 2D and 3D numerical simulation. They found that the lower temperature of TiAlN-coated tools would reduce the tensile residual stress of the machined workpiece. The above finding show that numerical simulation has high prediction accuracy and offers advantages in solving various cutting process parameters simultaneously. However, the problem of efficiency is still a shortcoming that is difficult to solve.

In recent years, with the continuous improvement and development of machine learning theory, a variety of methods based on mature machine learning theory have also been introduced into residual stress prediction and processing parameter optimization research, greatly improving models’ prediction accuracy [16]. Based on Gaussian process regression, Chen et al. [17] established a surface residual stress prediction model for the end milling process. They found that the feed rate had the greatest effect on surface residual stresses. Elsheikh et al. [18] predicted the residual stress induced by cutting IN718 alloy using a hybrid learning model of the pigeon optimization algorithm and particle swarm optimization. The effect of the cutting conditions on the induced relative errors was investigated. Jafarian [19] used an artificial neural network method to determine the optimal machining parameters to predict residual stress induced by finishing Inconel 718. The validation experiments showed that the prediction accuracy of the proposed hybrid machine model was better than that of traditional methods. Xu et al. [20] introduced k-nearest neighbors and artificial neural network methods to predict the effects of different cutting parameters on surface residual stress during high-speed milling. They concluded that the feed rate is the most important factor affecting the residual stresses.

The understanding of the above works has allowed for the prediction of residual stresses and a deeper understanding of the mechanisms of residual stresses using analytical and numerical models. In addition, residual stress prediction through intelligent optimization algorithms is a new trend. However, there is currently less literature on the prediction of residual stress induced by multi-axis milling.

In this study, an RBF neural network model based on simulated and experimental data is developed to predict the surface residual stress for multi-axis milling of Ti-6Al-4V titanium alloy. Firstly, a 3D numerical model is established for predicting surface residual stress in Ti-6Al-4V titanium alloy subjected to ball-end milling. Experiments in multi-axis milling and subsequent X-ray diffraction measurements are conducted, and the basic data of residual stress under various machining parameters are obtained via experimentation and simulation. Secondly, the RBF neural network is introduced to relate machining parameters and the surface residual stress. Finally, the interaction effect of machining parameters on surface residual stress is analyzed. The optimal conditions can be obtained to control the surface residual stress in the multi-axis milling of Ti-6Al-4V titanium alloy.

## 2. Numerical Model for Residual Stress Induced by Multi-Axis Milling

### 2.1. Simulation Details

**Figure 1:**
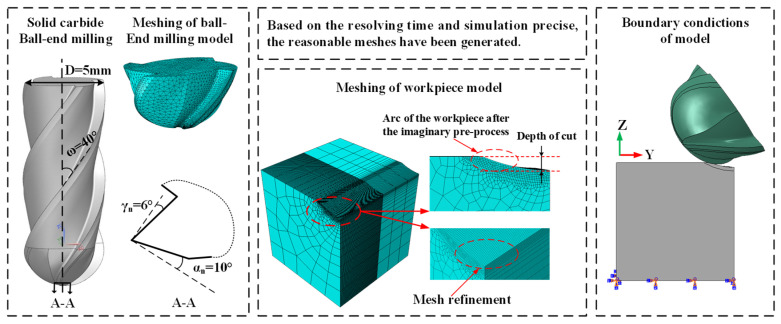
Numerical model for multi-axis milling of Ti-6Al-4V alloy.

A 3D numerical model of multi-axis milling of Ti-6Al-4V titanium alloy was developed using the explicit time integration method, and a coupled thermomechanical transient analysis was performed. The numerical model was developed using ABAQUS software. The initial boundary conditions and mesh settings of the numerical model of multi-axis milling are shown in Figure 1. The tool diameter is 5 mm, the rake angle is 6°, the relief angle is 10°, and the helix angle is 40°. The workpiece is a cube of 6 mm × 6 mm × 6 mm, and the cutting depth is 0.2 mm. To simulate the stable cutting stage, the workpiece was geometrically modified to establish a cutting area, and a 0.2 mm arc-shaped cutting depth was formed according to the tool size. The *x*-, *y*-, and *z*-directions are fixed at the bottom of the workpiece. The translation speed of the tool in the *y*-direction simulates the actual feed, and the rotation speed of the tool axis is given to simulate the rotation of the spindle.

To improve the authenticity and accuracy of the simulation, the initial temperature field was set for the workpiece and the tool, and the value was 25 °C. General contact mode was set, defined at the cutting area of the tool and the workpiece. The tool–workpiece–chip frictions followed Coulomb’s friction law, and the friction coefficient was set as 0.24 [21]. The heat generated by friction is equally distributed to the tool and the workpiece. Due to the irregular shape of the tool, the local seed distribution was selected for the cutting edge, and the characteristic length was 0.05 mm. The mesh type was selected as the temperature–displacement coupled tetrahedral element (C3D4T). The workpiece was meshed by a temperature–displacement coupled hexahedron, and the reduced integration mode (C3D8RT) was selected to improve the simulation efficiency without affecting the accuracy of the results [11]. The cutting area of the workpiece was geometrically divided, and the mesh was locally refined to reduce the number of elements. The characteristic length was 0.025 mm.

### 2.2. Governing Equations

To simulate the residual stress induced by the multi-axis milling process, it is necessary to solve the nonlinear spatial variation control equations in the region of the given boundary conditions and initial conditions. Equations (1)–(6) list a set of governing equations that needed to be solved in the present work [22]:

(1) $$\nabla *\overset{\cdot }{\sigma }=0$$

(2) $$\overset{\cdot }{\varepsilon }-\nabla^{s}V=0$$

(3) $$\sigma^{\nabla J}=D{\overset{\cdot }{\varepsilon }}^{el}=D(\overset{\cdot }{\varepsilon }-{\overset{\cdot }{\varepsilon }}^{vp}-{\overset{\cdot }{\varepsilon }}^{th})$$

(4) $${\overset{\cdot }{\varepsilon }}^{vp}=\left\|{\overset{\cdot }{\varepsilon }}^{vp}\right\|\frac{dev\left(\sigma \right)}{\left\|dev(\sigma )\right\|}=\left\|{\overset{\cdot }{\varepsilon }}^{vp}\right\|\overset{\wedge }{n}$$

(5) $$\sqrt{\frac{3}{2}}\left\|dev(\sigma )\right\|-\bar{\sigma }\geq 0$$

(6) $$K\nabla^{2}T+\frac{Dq_{gen}}{Dt}=\rho c\frac{DT}{Dt}$$

where Equation (1) is the equilibrium equation, and the terms ∇ and $\overset{\cdot }{\sigma }$ are the gradient operator and stress rate, respectively. Equation (2) is the strain rate ($\overset{\cdot }{\varepsilon }$) equation based on velocity (*V*), where $\nabla^{s}$ represents the velocity field. Equation (3) gives the relationship between the Jaumann stress rate and the strain rate of Cauchy stress. The parameter *D* is a material property matrix, while ${\overset{\cdot }{\varepsilon }}^{el}$, ${\overset{\cdot }{\varepsilon }}^{vp}$, and ${\overset{\cdot }{\varepsilon }}^{th}$ are the elastic strain, viscoplastic strain rate, and thermal strain rate, respectively. Equation (4) is the flow rule, which gives the viscoplastic flow direction of the ideal plasticity theory, and the occurrence condition is determined by the yield criterion in Equation (5). Equation (6) represents the heat equation, where the parameters *K*, ρ, *c*, and $Dq_{gen}/Dt$ are the thermal conductivity, density, specific heat, and heat generation rate, respectively.

Metal cutting is a complex thermomechanical coupling process that involves many basic theoretical disciplines, such as elastoplastic mechanics, thermodynamics, fracture mechanics, and tribology. The constitutive model of the material determines the results of force and stress. The chip separation criteria determine whether the chip can be generated correctly. Considering the influence of various factors, the widely recognized Johnson-Cook model expressed in Equation (7) is used to characterize the constitutive relationship, and the Johnson-Cook dynamic failure model expressed in Equation (8) is used as the chip separation criterion.

(7) $$\sigma =\left[A+B\varepsilon^{n}\right]\left[1+C\ln\frac{\dot{\varepsilon }}{{\dot{\varepsilon }}_{0}}\right]\left[1-{\left(\frac{T-T_{r}}{T_{m}-T_{r}}\right)}^{m}\right]$$

(8) $$\bar{\varepsilon^{f}}=\left[d_{1}+d_{2}\exp\left(d_{3}\frac{P}{\overset{-}{\sigma }}\right)\right]\left[1+d_{4}\ln\left(\frac{\overset{\cdot }{\overset{-}{\varepsilon }}}{\overset{\cdot }{\varepsilon_{0}}}\right)\right]\left[1+d_{5}\left(\frac{T-T_{r}}{T_{m}-T_{r}}\right)\right]$$

where the flow stress (*σ*) of the material is expressed by the strain (*ε*), strain rate ($\overset{\cdot }{\varepsilon }$), and temperature (*T*); $\overset{\cdot }{\varepsilon_{0}}$ is the equivalent strain rate, *T* is the material temperature of the workpiece, *T_r_* is the room temperature, and *T_m_* is the melting temperature. In Equation (7), the constants A, B, and C are the initial yield stress, hardening modulus, and strain rate variation coefficient, respectively, while the constants m and n represent the thermal softening coefficient and work hardening index, respectively. In Equation (8), *d*_1_, *d*_2_, and *d*_3_ are the strain-dependent damage parameters. The parameters *d*_4_ and *d*_5_ represent the strain-rate-dependent damage parameter and the thermal softening parameter, respectively. The Johnson–Cook constitutive model parameters and damage model parameters for Ti-6Al-4V titanium alloy are listed in Table 1 and Table 2, respectively. The thermomechanical properties of the Ti-6Al-4V titanium alloy are shown in Table 3.

### 2.3. Data Extraction of Simulated Residual Stress

**Figure 2:**
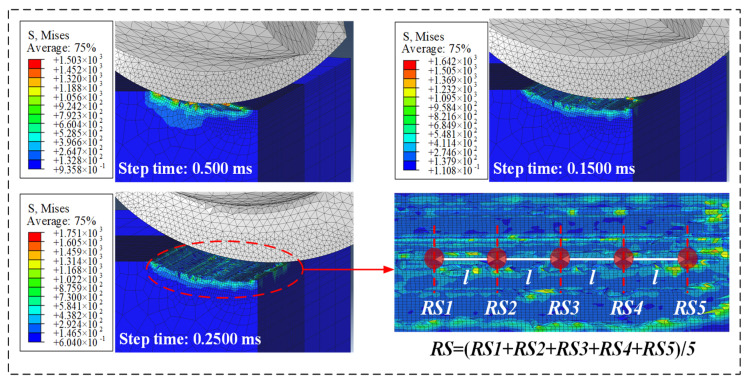
Simulation results and data extraction.

The simulation of residual stress can be divided into four basic processes: Firstly, the cutter performs the cutting motion according to the given cutting parameters to complete the milling process. Secondly, the tool is retreated to eliminate the influence of cutting force on the stress and strain of the workpiece. Thirdly, the fixed constraint at the bottom of the workpiece is removed and transformed into a three-point constraint, so that the workpiece can deform freely. Finally, the workpiece is set with thermal convection contact to complete cooling. Residual stress is unevenly distributed on the workpiece due to discontinuous and continuous changes in the cutting point. Therefore, five test points (RS1, RS2, RS3, RS4, and RS5) of the machined surface in Figure 2 are selected to obtain the average distribution of surface residual stress. 

### 2.4. Verification Experiment

#### 2.4.1. Machining Experiment

**Figure 3:**
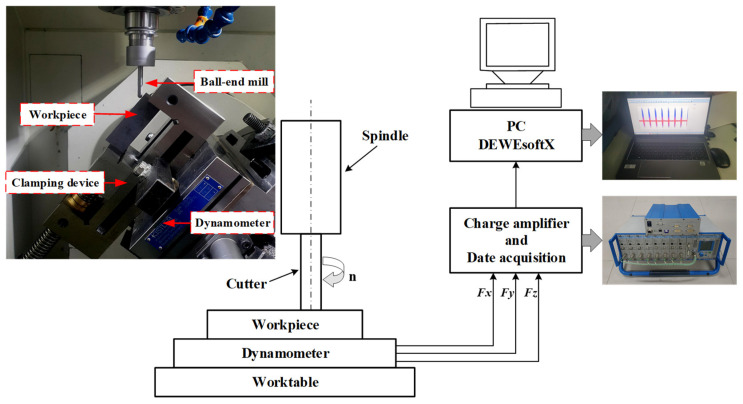
Schematic diagram of the multi-axis milling experiment.

The experiments of multi-axis milling of Ti-6Al-4V titanium alloy were conducted on a five-axis vertical NC (JingDiao, Beijing, China)under dry cutting conditions. The milling method was climb cutting. The workpiece was machined into a rectangular block with dimensions of 60 mm × 40 mm × 25 mm. The workpiece was annealed and stress-relieved in the furnace before the experiment. The surface residual stress of the treated workpiece was −32.8 ± 12.5 MPa in the σ*_x_* direction and −27.6 ± 15.4 MPa in the σ*_y_* direction. To measure the initial residual stress inside the workpiece, the workpiece was electropolished with an electropolishing instrument (SOLY, Shanghai, China), and the polishing was stopped when the residual stress value approached zero. The residual stress was approximately zero when the depth was 80 μm (−7.6 ± 13.9 MPa in the σ*_x_* direction and 15.1 ± 20.3 MPa in the σ*_y_* direction). The tool used in the experiment was a four-tooth ball-end mill of R2.5 mm × 25 mm × 65 mm. The tool material was carbide (K 44) and uncoated. Ball-end milling with a rake angle of 6°, relief angle of 10°, and helix angle of 40° is often adopted. The overhang length was fixed as 35 mm. The machining parameters in all experiments were fixed at an axial milling depth of a_p_ = 0.2 mm and a radial milling depth of a_e_ = 0.2 mm. Additionally, a new cutting edge was employed to avoid the effect of cutter wear on the cutting edge for each experiment. The selection of machined parameters mainly refers to the parameters in the actual production of the blades. There are three design variables: the inclination angle (*θ*), the cutting speed (*V_c_*), and the feed rate (*f*_z_). The cutting force was measured by a three-way dynamic piezoelectric dynamometer, which was connected to the computer via a charge amplifier and data acquisition. In the machine coordinate system, the force components are defined as main force *Fx* (N), feed force *Fy* (N), and radial force *Fz* (N). The cutting force signal was collected using Dewesoftx software. The multi-axis milling experiment and cutting force measurements are shown in Figure 3.

#### 2.4.2. Residual Stress Measurement

**Figure 4:**
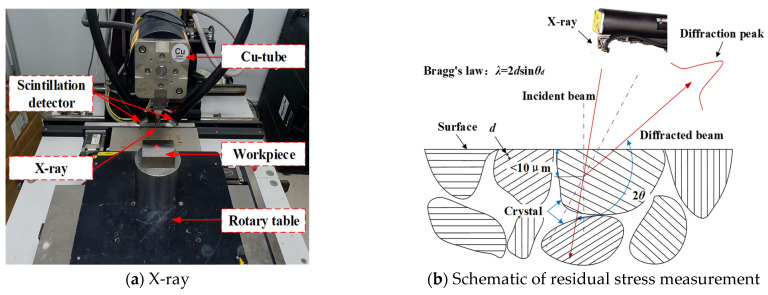
Residual stress measurement.

Due to the relationship between the macroscopic residual stress and the final performance of the component, only the macroscopic residual stress is studied in this paper. This is considered to be one of the main factors affecting machining accuracy, dimensional stability, and product life. The LXRD MG2000 X-ray residual stress testing system (PROTO, Toronto, Canada)was used to measure the residual stress of the specimens. Cu K-Alpha radiation was used in the residual stress test with the sin2W method. The test voltage was 25 kV and the electric current was 30 mA. A circular spot with a diameter of 3 mm was used. The exposure time was 2 s, and the number of exposures was 10. The Bragg angle was set to 142°, and the swing range of the β angle was ±25°. In order to ensure the validity of the residual stress test results, each point was measured three times, and the average value was calculated until the error was within the allowable range. The secondary stress measurements were performed using a turntable, as shown in Figure 4a. The X-ray stress measurements were based on crystal Bragg diffraction. When monochromatic X-rays were incident on the polycrystal shown in Figure 4b, the diffraction of the crystal occurred at a satisfactory angle:

*λ* = 2*d*sin*θ_d_* (9)

where *λ* is the wavelength of the X-ray, *d* is the interplanar spacing of the atomic plane, and *θ_d_* is the angle between the incident ray and the atomic plane, called the Bragg angle. The lattice plane {213} of Ti was used for the measurement. The cutting load causes changes in the lattice spacing on the workpiece surface. The stress-free value changes from *d*_0_ to $d_{0}+\Delta d$, and the strain in the normal direction of the diffraction plane is as follows:

(10) $$\varepsilon =\frac{\Delta d}{d_{0}}=-\cot\theta_{0}(\theta_{d}-\theta_{0})$$

where, $\theta_{0}$ is the diffraction angle of the unstressed crystal.

#### 2.4.3. Simulated and Measured Cutting Forces and Chip Morphology

**Figure 5:**
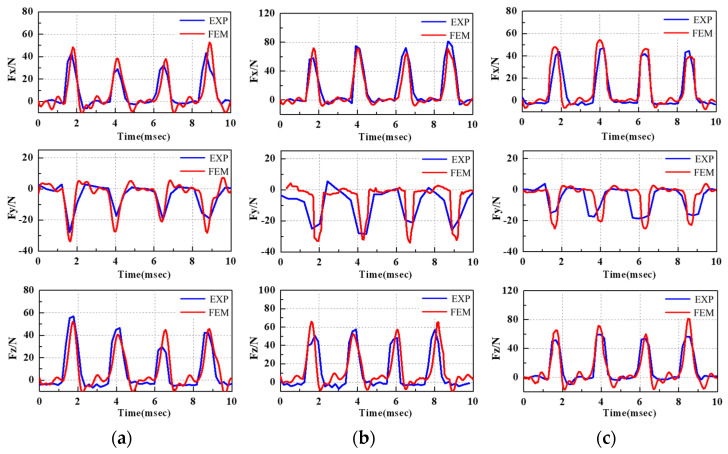
Experimental and simulated cutting forces: (**a**) *θ* = 35°, *V_c_* = 100 m/min, *f*_z_ = 0.05 mm/rev; (**b**) *θ* = 55°, *V_c_* = 100 m/min, *f*_z_ = 0.09 mm/rev; (**c**) *θ* = 75°, *V_c_* = 100 m/min, *f*_z_ = 0.03 mm/rev.

The consistency of simulated and experimental cutting force results can improve the reliability of residual stress prediction. Figure 5 displays the filtered cutting force comparison between the simulated results and those measured by dynamometer. Ball-end milling entails intermittent cutting. The collection frequency of cutting force depends on the spindle speed and the number of cutting edges. Since the tool has four edges, four peaks and valleys can be observed in *F_x_*, *F_y_*, and *F_z_*. It can be observed from the experimental and simulated cutting forces that the variation ranges for cutting force per cutting edge are small, and the milling process is stable. The error rates of the maximum cutting force of *Fx* in the three groups of machining parameters are 12.7%, 6.4%, and 4.8%, respectively; the average error is 7.9%. The error rates of the maximum cutting force of *Fy* in the three groups of machining parameters are 11.5%, 14.8%, and 10.3%, respectively; the average error is 12.2%. The error rates of the maximum cutting force of *Fz* in the three groups of machining parameters are 5.4%, 13.7%, and 8.6%, respectively; the average error rate is 9.2%. The results show that the established numerical model has high prediction accuracy for cutting force.

**Figure 6:**
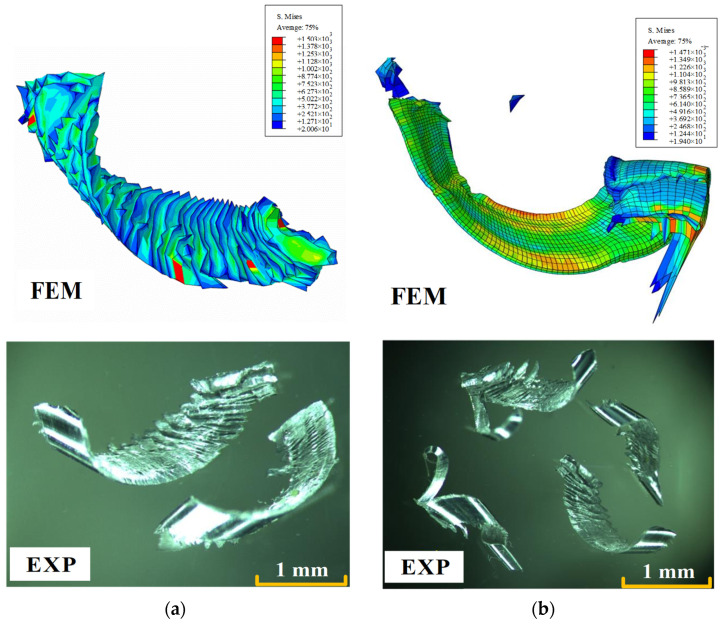
Experimental and simulated chip morphology: (**a**) *θ* = 35°, *V_c_* = 20 m/min, *f*_z_ = 0.07 mm/rev; (**b**) *θ* = 55°, *V_c_* = 20m/min, *f*_z_ = 0.03 mm/rev.

In addition to cutting forces, chip formation is also very important for the study of various complex machining processes. The comparison of chip morphology between simulated and experimental results is shown in Figure 6. It can be seen that both the simulated and experimental chip surfaces have serrated wrinkles, and that the degree of spatial curling is similar. The reason for this is that the high friction between the tool and the chip leads to excessive temperature in the cutting zone, resulting in a large deformation of the chip as it flows over the rake face. The good consistency between the wrinkled and curled chips obtained by numerical simulation and the real experiments can prove that the actual milling process is balanced and stable. The similarity of chip formation can also directly reflect the accuracy and effectiveness of the numerical model.

#### 2.4.4. Simulated and Measured Surface Residual Stress

**Figure 7:**
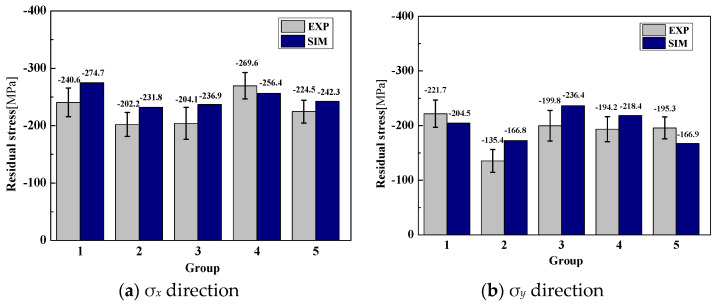
Experimental and simulated surface residual stress.

Figure 7 illustrates the comparison of simulated and experimental surface residual stress in the σ*_x_* and σ*_y_* directions under the various machining parameters. The simulated surface residual stress is consistent with the experimental values, reflecting the variation of residual stress in two directions. Both the simulated and experimental results show that the surface residual stress is compressive stress. The experimental and simulated values and errors of the surface residual stress of the five groups of machining parameters are shown in Table 4. It can be seen that the experimental surface residual stress ranges from −202.2 MPa to −269.6 MPa in the σ*_x_* direction, and from −135.4 MPa to −221.7 MPa in the σ*_y_* direction. The established numerical model has a maximum prediction error rate of 16.1% and a minimum error rate of 4.9% in the σ*_x_* direction, and a maximum prediction error rate of 23.2% and a minimum error rate of 7.8% in the σ*_y_* direction. The total average prediction error rate of surface residual stress is 11.6% and 15.2% in the two directions, respectively. The average error is less than 20%, showing that the established 3D numerical model can better predict the surface residual stress of multi-axis milling.

## 3. RBF Neural Network Model of Surface Residual Stress

### 3.1. RBF Neural Network

**Figure 8:**
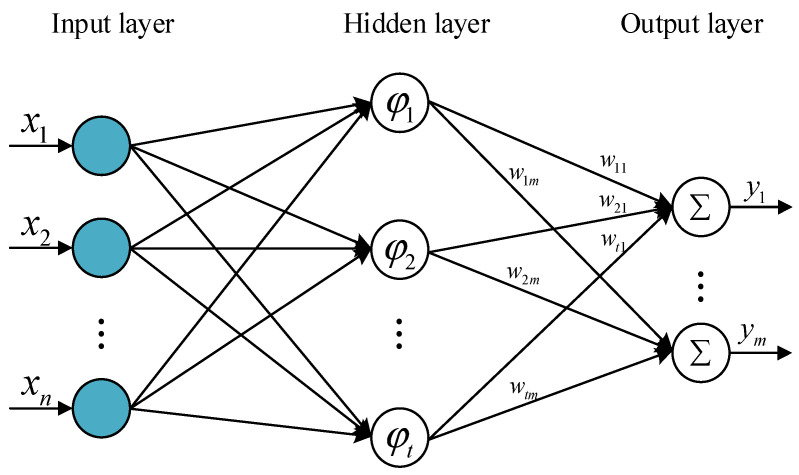
RBF neural network structure.

An RBF neural network is a forward network with a single hidden layer, which can be used for function approximation and model prediction [26]. RBF neural networks are good at modeling nonlinear data, which can be trained in one stage and can also quickly learn a given application. The basic RBF neural network structure includes an input layer, a hidden layer, and an output layer. The input layer consists of some perceptual units that connect the network with the external environment. The RBF network has only one hidden layer, which nonlinearly transforms the input vector from the input space to the hidden space. The output of the network is a linear weight of the hidden units, as shown in Figure 8. The hidden layer can be linearly combined with the output layer, and the processing speed is extremely rapid [27]. The mathematical definition of the hidden neuron is shown in Equation (11):

(11) $$\varphi =(\left|x-x_{j}\right|),j=1,2,\ldots ,n$$

The neurons in the input layer are directly connected to the neurons in the hidden layer. The hidden layer contains multiple kernel functions $\varphi_{j}(x)$, which contain a center of the j-th input data point $x_{j}$ and width $\varphi_{j}$(spread).

The Gaussian function is used as the basis function; it is defined in Equation (12):

(12) $$\varphi_{j}(x)=\exp(-1/2\sigma_{i}^{2}{\left(\left\|x-x_{j}\right\|\right)}^{2}),j=1,2,\ldots ,n$$

where *x* represents the n-dimensional input vector, while *σ*_i_ is the i-th perceptual variable, which determines the width of the basis function around the center point. The size of the $x_{j}$ value reflects the response width of the output to the input. The input of the output layer is the weighted summation of the outputs of the neurons in the hidden layers. Since the excitation function is a purely linear function, it is defined in Equation (13):

(13) $$F(x)=\sum_{j=1}^{n}\omega_{j}\varphi_{j}(x)$$

where $\omega_{j}$ represents the output weights. The weights of only a few output layers need to be adjusted and determined during the RBF network training process. The RBF neural network has optimal approximation and global optimal performance, meaning that it does not easily converge to the local optimal solution, and the network training needs to adjust fewer parameters. Therefore, RBF neural networks have been widely used in solving nonlinear problems.

### 3.2. Prediction Model of Surface Residual Stress

**Figure 9:**
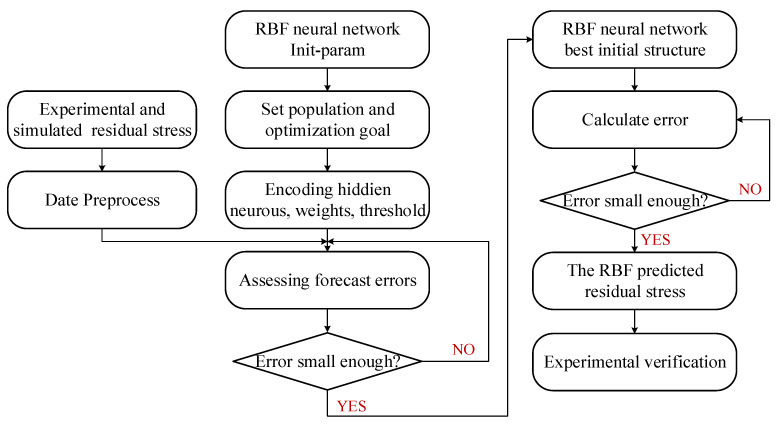
Flowchart of the prediction model based on an RBF neural network.

Residual stress is very sensitive to machining parameters, and milling is a nonlinear large-deformation process. In theory, RBF neural networks are suitable for this engineering problem. The three input variables in this paper are the inclination angle (*θ*), the cutting speed (*V_c_*), and the feed rate (*f_z_*). The output layer is the surface residual stress value in both the σ_x_ and σ_y_ directions. The input vector is *x* = [*θ*, *V_c_*, *f_z_*]. The dimensions of the three are not consistent, so the input vector is normalized.

(14) $$x_{i}=(x-x_{i}^{\min})/(x_{i}^{\max}-x_{i}^{\min})$$

where *x_i_* is the i-th component of the input vector, while $x_{i}^{max}$ and $x_{i}^{min}$ are the maximum and minimum values of the i-th component, respectively. The RBF model was established through the MATLAB platform. The flowchart of the prediction model based on the RBF neural network is shown in Figure 9.

**Figure 10:**
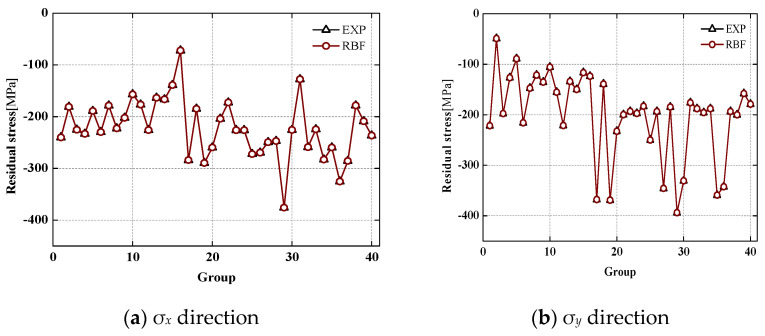
Experimental and predicted coefficients with the trained RBF network.

The spread parameter was 0.42. The 40 groups of experimental and simulation data were used as training samples, as shown in Table 5, to train the network and obtain the optimal network structure. Five additional experiments were selected within the design parameter threshold to verify the accuracy of the RBF model, and the experimental conditions remained unchanged. Therefore, the number of hidden layer neurons was only 40 for the residual stresses in each direction. The index of error evaluation is defined as shown in Equation (15):

(15) $$e=\frac{\sqrt{{\left(\sum_{i}^{I}\chi_{i}^{pre}-\chi_{i}\right)}^{2}/I}}{\left(\sum_{i}^{I}\left|\chi_{i}\right|\right)/I}$$

where *e* is the average error, $\chi_{i}^{pre}$ is the predicted value of the RBF neural network, $\chi_{i}$ is the experimental or simulated value, and I is the total number of experiments and simulations. Figure 10 shows the comparison between the fitting value of the trained RBF neural network and the experimental value. It can be seen that the fitting value of the RBF neural network at the experimental point almost completely coincides with the experimental result. This indicates that the RBF model has completed the best approximation to the sample point.

### 3.3. Verification of Residual Stress

**Figure 11:**
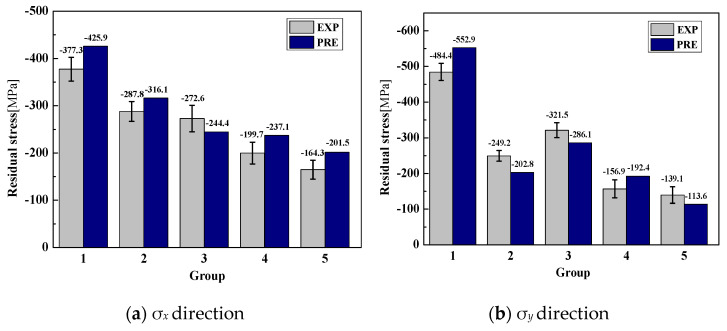
Experimental and predicted surface residual stress.

To evaluate the applicability of the designed neural network, five groups of machining parameters within the design parameter threshold were selected for verification experiments. The experimental conditions remained unchanged. The comparison of the predicted surface residual stress and the corresponding experimental results is shown in Figure 11. Clearly, the predicted values are consistent with the experimental values for all five groups of machining parameters. The surface residual stress is residual compressive stress. The experimental and predicted values and errors of the surface residual stress of the five groups of machining parameters are shown in Table 6. It can be observed that the error range of the experimental and predicted values for the five groups of machining parameters is 28.2 MPa ~ 48.6 MPa in the σ*_x_* direction; the error rates are 12.9%, 9.8%, 10.4%, 16.2%, and 22.7%, respectively. The average prediction error of surface residual stress is 14.4%. The prediction error range is between 25.5 MPa ~ 68.5 Mpa in the σ*_y_* direction, and the error rates are 14.2%, 18.7%, 11.1%, 22.6%, and 19.3%, respectively. The average prediction error of surface residual stress is 17.2%. The prediction error of the surface residual stress in the σ*_x_* direction is smaller than that in the σ*_y_* direction. Meanwhile, the error of the established prediction model is slightly higher than that of the simulation model. The reason for this is that a large number of simulation samples are selected in the prediction model, and there is a certain deviation between the simulation and the experiment. Overall, this validation shows that surface residual stress can be well predicted using the modeling method presented in this paper.

## 4. Influence of Machining Parameters 

Cutting is a highly nonlinear, high-temperature, and large-deformation elastoplastic behavior [28]. The thermal-related properties of the material are in an unstable state. Meanwhile, the thermomechanical coupling effect causes alternating fluctuations of thermal stress and mechanical stress inside the material, which is a typical material nonlinear and geometric nonlinear problem [29]. The mechanical behavior of tool-workpiece contact is also in a time-varying state during the cutting process. For the stress solution inside the surface material of the workpiece, the time-varying characteristics of the contact boundary make the problem more complicated, which is a typical nonlinear boundary condition problem. Hence, there may be a high degree of nonlinearity between the machining parameters and the cutting response, and it is difficult to obtain a linear mapping by simply transforming the sample data sequence.

**Figure 12:**
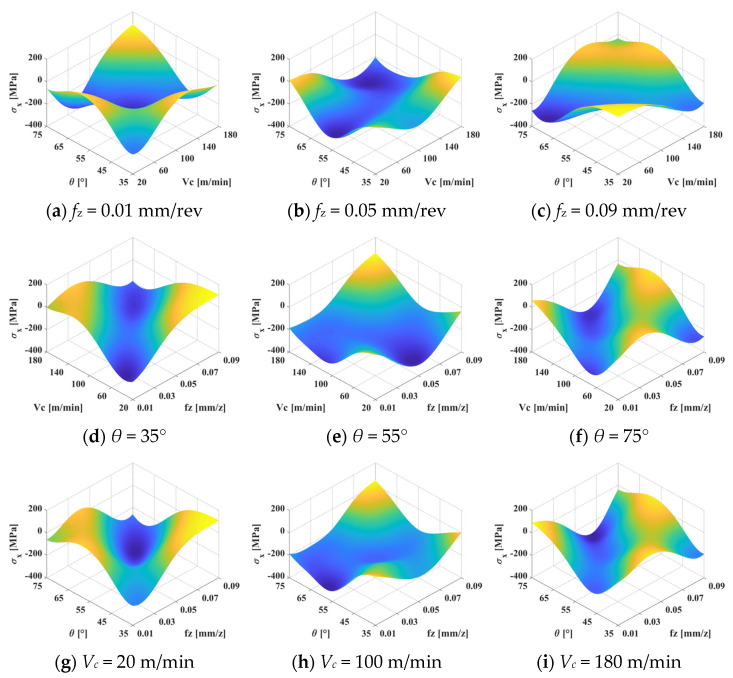
Interactive effects of machining parameters on surface residual stress in the σ*_x_* direction.

**Figure 13:**
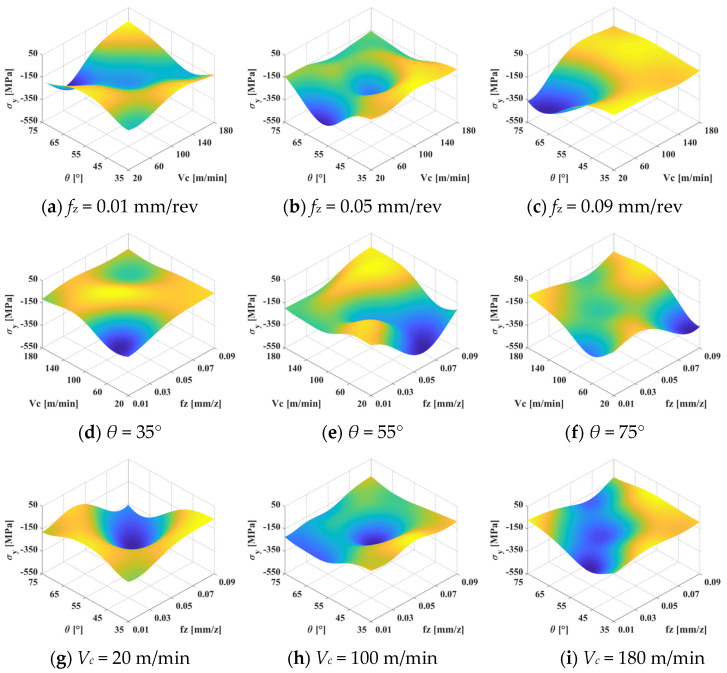
Interactive effects of machining parameters on surface residual stress in the σ*_y_* direction.

Figure 12 and Figure 13 show the interactive effects of the machining parameters on surface residual stress in the σ*_x_* and σ*_y_* directions, respectively. It can be seen that the influence of the inclination angle, milling speed, and feed rate on surface residual stress is strongly nonlinear. In Figure 12a–c, the maximum value of residual compressive stress is always located in the middle parameter region in the σ*_x_* direction. Similar laws also apply in the σ*_y_* direction when the feed rate is small, as shown in Figure 13a. Parameters that are too large or too small will lead to the transformation of the residual stress form from compression to tension. The residual tensile stress first decreases and then increases with increasing feed rate. In the σ*_y_* direction, the cutting speed and inclination angle have little effect on the residual stress when the feed rate exceeds 0.05 mm/rev. A lower cutting speed and larger inclination angle can lead to larger residual compressive stress, as shown in Figure 13b,c. Therefore, a large residual compressive stress can be obtained at an inclination angle of 55° and a cutting speed of about 100 m/min.

In Figure 12d–f and Figure 13d–f, it can be observed that the variation law of residual stress is relatively similar in the two directions. The low cutting speed and feed rate can generate a large residual compressive stress when the inclination angle is 35°, which is about −300 MPa in the σ*_x_* direction and −450 MPa in the σ*_y_* direction. As the inclination angle increases from 35° to 55°, the maximum residual tensile stress moves from a small feed rate to a large feed rate. The maximum value is located in the area where the feed rate is higher than 0.05 mm/rev, and the maximum residual stress first increases and then decreases. However, the trend is the opposite when the inclination angle increases to 75°. This confirms that an inclination angle that is too large or too small will lead to a reduction in residual compressive stress. Overall, a large residual compressive stress can be obtained when the inclination angle is 55°. The maximum residual compressive stress is obtained in the neighborhood with a cutting speed of 100 m/min and a feed rate of 0.05 mm/rev.

The interactive effects of the feed rate and inclination angle on surface residual stress are shown in Figure 12g–i and Figure 13g–i. The maximum residual compressive stress is located at the small feed rate threshold. A small inclination angle can produce a large residual compressive stress when the cutting speed is slow. However, a large residual compressive stress can be obtained by selecting the inclination angle near the neighborhood of 55° when the cutting speed is high. The residual compressive stress has a trend of first increasing and then decreasing with the increase in cutting speed. To intuitively show the optimal region of residual stress, the color is used to describe the value of residual stress. It can be seen that the maximum value of global compressive stress is located in the mazarine area.

## 5. Conclusions

In the present study, for the purpose of predicting the surface residual stress induced by multi-axis milling of Ti-6Al-4V titanium alloy, a new prediction model is proposed, combining simulation and experimentation. The experimental results verify the accuracy of the established model. Some conclusions can be summarized as follows:

(1) A 3D numerical model was established to predict the surface residual stress induced by multi-axis milling of Ti-6Al-4V titanium alloy. The simulated and experimental values followed a similar trend and gave good agreement between them. The total average prediction error rate of surface residual stress was 11.6% and 15.2% in the σ*_x_* and σ*_y_* directions, respectively.
(2) The verification shows that the surface residual stress can be well predicted by using the modeling method based on the RBF neural network presented in this paper. Within the thresholds of cutting speed of 20~180 m/min, feed rate of 0.01~0.09 mm/rev, and roll angle of 35~75°, the prediction error ranges from 28.2 MPa to 48.6 MPa in the σ*_x_* direction, and from 25.5 MPa to 68.5 MPa in the σ*_y_* directions. The average prediction error rates of surface residual stress in the two directions are 14.4% and 17.2%, respectively. The proposed prediction model in this paper, based on experimentation and simulation, can effectively predict the surface residual stress induced by multi-axis milling of Ti-6Al-4V titanium alloy.
(3) The inclination angle, cutting speed, and feed rate have a strong nonlinear relationship with the surface residual stress. Parameters that are too large or too small will lead to the transformation of the surface residual stress form from compression to tension.

## Figures and Tables

**Table 1:** Johnson–Cook constitutive model parameters of Ti-6Al-4V [23].

A	B	C	*n*	*m*	${\overset{\overset{\cdot }{-}}{\varepsilon }}_{0}$
782	498	0.028	0.28	1	0.0001

**Table 2:** Johnson–Cook damage model parameters of Ti-6Al-4V [24].

*d*_1_	*d*_2_	*d*_3_	*d*_4_	*d*_5_
−0.09	0.25	−0.5	0.014	3.87

**Table 3:** Thermomechanical properties of titanium alloy Ti-6Al-4V [25].

Elastic Modulus (MPa)	Poisson’s Ratio	Heat Capacity (J/(Kg °C))	Conductivity (W/(m °C))	Expansion (μm/(m °C))
0.7412 T + 113375	0.34	2.24 e^0.0007T^	7.039 e^0.0011T^	3 × 10^−9^ T + 7 × 10^−6^

**Table 4:** Experimental and simulated surface residual stress.

No.	*θ* (°)	*V_c_* (m/min)	*f*_z_ (mm/rev)	σ*_x_* (MPa)			σ*_y_* (MPa)		
				EXP	SIM	Error	EXP	SIM	Error
1	35	60	0.03	−240.6	−274.7	14.2%	−221.7	−204.5	7.8%
2	45	100	0.07	−202.2	−231.8	14.7%	−135.4	−166.8	23.2%
3	55	180	0.05	−204.1	−236.9	16.1%	−199.8	−236.4	18.3%
4	65	140	0.03	−269.6	−256.4	4.9%	−194.2	−218.4	12.5%
5	75	100	0.03	−224.5	−242.3	8.1%	−195.3	−166.9	14.4%

**Table 5:** Sample data of surface residual stress.

No.	*θ* (°)	*V_c_* (m/min)	*f*_z_ (mm/rev)	σ*_x_* (MPa)	σ*_y_* (MPa)	Source
1	35	60	0.03	−240.16	−221.7	EXP
2	35	100	0.05	−181.1	−49.1	EXP
3	35	20	0.01	−225.3	−197.6	SIM
4	35	140	0.07	−233.1	−126.8	SIM
5	35	180	0.09	−189	−89.1	SIM
6	35	60	0.07	−229.7	−215.9	SIM
7	35	100	0.03	−178.6	−147.5	SIM
8	35	140	0.03	−222.7	−121.6	SIM
9	45	100	0.07	−202.2	−135.4	EXP
10	45	140	0.09	−157	−105.6	EXP
11	45	20	0.03	−176.61	−155.3	SIM
12	45	180	0.01	−226	−221.3	SIM
13	45	100	0.05	−163.5	−134.1	SIM
14	45	20	0.07	−166.6	−149.9	SIM
15	45	60	0.09	−138.9	−116.6	SIM
16	45	100	0.01	−72.1	−123.7	SIM
17	55	60	0.07	−284	−367.7	EXP
18	55	100	0.09	−184.7	−139.1	EXP
19	55	20	0.05	−289.5	−369	SIM
20	55	140	0.01	−259.6	−232.6	SIM
21	55	180	0.03	−204.1	−199.8	SIM
22	55	180	0.05	−172.6	−193.4	SIM
23	55	100	0.07	−226.1	−197.4	SIM
24	55	100	0.03	−226.1	−183.1	SIM
25	65	100	0.01	−272.3	−250	EXP
26	65	140	0.03	−269.6	−193.5	EXP
27	65	60	0.09	−249.3	−345.6	SIM
28	65	180	0.05	−247	−184.6	SIM
29	65	20	0.03	−375.8	−393.7	SIM
30	65	60	0.03	−225.7	−330.7	SIM
31	65	100	0.09	−127.8	−176.2	SIM
32	65	140	0.07	−258.9	−187.9	SIM
33	75	100	0.03	−224.5	−195.8	EXP
34	75	140	0.05	−283	−187.9	EXP
35	75	20	0.09	−259.5	−359	SIM
36	75	60	0.01	−325.3	−342.4	SIM
37	75	180	0.07	−285.6	−193.5	SIM
38	75	100	0.05	−178.4	−200.1	SIM
39	75	140	0.03	−208.9	−157.9	SIM
40	75	180	0.07	−236.5	−179.1	SIM

**Table 6:** Experimental and predicted surface residual stress.

No.	*θ* (°)	*V_c_* (m/min)	*f*_z_ (mm/rev)	σ*_x_* (MPa)			σ*_y_* (MPa)		
				EXP	PRE	Error	EXP	PRE	Error
1	65	20	0.07	−377.3	−425.9	12.9%	−484.4	−552.9	14.2%
2	55	100	0.01	−287.8	−316.1	9.8%	−249.2	−202.8	18.7%
3	55	100	0.05	−272.6	−244.4	10.4%	−321.5	−286.1	11.1%
4	65	100	0.05	−199.7	−232.1	16.2%	−156.9	−192.4	22.6%
5	45	60	0.05	−164.3	−201.5	22.7%	−139.1	−113.6	19.3%

## Data Availability

Not applicable.

## References

[1] Mirzendehdel A.M., Behandish M., Nelaturi S. (2020). Topology optimization with accessibility constraint for multi-axis machining. Comput.-Aided Des..

[2] Wan M., Ye X.-Y., Wen D.-Y., Zhang W. (2019). Modeling of machining-induced residual stresses. J. Mater. Sci..

[3] Wu G., Li G., Pan W., Raja I., Wang X., Ding S. (2021). A state-of-art review on chatter and geometric errors in thin-wall machining processes. J. Manuf. Process..

[4] Yue C., Gao H., Liu X., Liang S.Y. (2018). Part functionality alterations induced by changes of surface integrity in metal milling process: A review. Appl. Sci..

[5] Li B., Deng H., Hui D., Hu Z., Zhang W. (2020). A semi-analytical model for predicting the machining deformation of thin-walled parts considering machining-induced and blank initial residual stress. Int. J. Adv. Manuf. Technol..

[6] Gao H., Zhang Y., Wu Q., Li B. (2018). Investigation on influences of initial residual stress on thin-walled part machining deformation based on a semi-analytical model. J. Mater. Process. Technol..

[7] Liang X., Liu Z., Wang B., Song Q., Cai Y., Wan Y. (2021). Prediction of residual stress with multi-physics model for orthogonal cutting Ti-6Al-4V under various tool wear morphologies. J. Mater. Process. Technol..

[8] Ji X., Liang S.Y. (2017). Model-based sensitivity analysis of machining-induced residual stress under minimum quantity lubrication. Proc. Inst. Mech. Eng. Part B J. Eng. Manuf..

[9] Ji X., Zhang X., Liang S.Y. (2014). Predictive modeling of residual stress in minimum quantity lubrication machining. Int. J. Adv. Manuf. Technol..

[10] Outeiro J., Umbrello D., M’saoubi R. (2006). Experimental and numerical modelling of the residual stresses induced in orthogonal cutting of AISI 316L steel. Int. J. Mach. Tools Manuf..

[11] Arrazola P.J., Kortabarria A., Madariaga A., Esnaola J., Fernandez E., Cappellini C., Ulutan D., Özel T. (2014). On the machining induced residual stresses in IN718 nickel-based alloy: Experiments and predictions with finite element simulation. Simul. Model. Pract. Theory.

[12] Xin H., Shi Y., Ning L., Zhao T. (2016). Residual stress and affected layer in disc milling of titanium alloy. Mater. Manuf. Process..

[13] Sahu N.K., Andhare A.B. (2019). Prediction of residual stress using RSM during turning of Ti–6Al–4V with the 3D FEM assist and experiments. SN Appl. Sci..

[14] Özel T., Ulutan D. (2012). Prediction of machining induced residual stresses in turning of titanium and nickel based alloys with experiments and finite element simulations. CIRP Ann..

[15] Mishra S.K., Ghosh S., Aravindan S. Finite element investigations on temperature and residual stresses during machining Ti6Al4V alloy using TiAlN coated plain and textured tools. Proceedings of the International Conference on Precision, Meso, Micro and Nano Engineering, COPEN.

[16] Ayeb M., Frija M., Fathallah R. (2019). Prediction of residual stress profile and optimization of surface conditions induced by laser shock peening process using artificial neural networks. Int. J. Adv. Manuf. Technol..

[17] Cheng M., Jiao L., Yan P., Feng L., Qiu T., Wang X., Zhang B. (2021). Prediction of surface residual stress in end milling with Gaussian process regression. Measurement.

[18] Elsheikh A.H., Muthuramalingam T., Shanmugan S., Ibrahim A.M.M., Ramesh B., Khoshaim A.B., Moustafa E.B., Bedairi B., Panchal H., Sathyamurthy R. (2021). Fine-tuned artificial intelligence model using pigeon optimizer for prediction of residual stresses during turning of Inconel 718. J. Mater. Res. Technol..

[19] Jafarian F., Amirabadi H., Fattahi M. (2014). Improving surface integrity in finish machining of Inconel 718 alloy using intelligent systems. Int. J. Adv. Manuf. Technol..

[20] Xu L., Huang C., Li C., Wang J., Liu H., Wang X. (2021). An improved case based reasoning method and its application in estimation of surface quality toward intelligent machining. J. Intell. Manuf..

[21] Ullah I., Zhang S., Waqar S. (2022). Numerical and experimental investigation on thermo-mechanically induced residual stress in high-speed milling of Ti-6Al-4V alloy. J. Manuf. Process..

[22] Ee K., Dillon O., Jawahir I. (2005). Finite element modeling of residual stresses in machining induced by cutting using a tool with finite edge radius. Int. J. Mech. Sci..

[23] Ren J., Cai J., Zhou J., Shi K., Li X. (2018). Inverse determination of improved constitutive equation for cutting titanium alloy Ti-6Al-4V based on finite element analysis. Int. J. Adv. Manuf. Technol..

[24] Wang Q., Liu Z., Yang D., Mohsan A.U.H. (2017). Metallurgical-based prediction of stress-temperature induced rapid heating and cooling phase transformations for high speed machining Ti-6Al-4V alloy. Mater. Des..

[25] Pan Z., Liang S.Y., Garmestani H., Shih D., Hoar E. (2019). Residual stress prediction based on MTS model during machining of Ti-6Al-4V. Proc. Inst. Mech. Eng. Part C J. Mech. Eng. Sci..

[26] Vahabli E., Rahmati S. (2016). Application of an RBF neural network for FDM parts’ surface roughness prediction for enhancing surface quality. Int. J. Precis. Eng. Manuf..

[27] Zhou J., Ren J., Tian W. (2017). Grey-RBF-FA method to optimize surface integrity for inclined end milling Inconel 718. Int. J. Adv. Manuf. Technol..

[28] Marek A., Davis F.M., Rossi M., Pierron F. (2019). Extension of the sensitivity-based virtual fields to large deformation anisotropic plasticity. Int. J. Mater. Form..

[29] Singh P., Pungotra H., Kalsi N.S. (2016). On the complexities in machining titanium alloys. CAD/CAM, Robotics and Factories of the Future.

